# Predictors of Maternal Death Among Women With Pulmonary Hypertension in China From 2012 to 2020: A Retrospective Single-Center Study

**DOI:** 10.3389/fcvm.2022.814557

**Published:** 2022-04-18

**Authors:** Ling-Ling Dai, Tian-Ci Jiang, Peng-Fei Li, Hua Shao, Xi Wang, Yu Wang, Liu-Qun Jia, Meng Liu, Lin An, Xiao-Gang Jing, Zhe Cheng

**Affiliations:** ^1^Department of Pulmonary and Critical Care Medicine, The First Affiliated Hospital of Zhengzhou University, Zhengzhou, China; ^2^Department of Anaesthesiology, Pain and Perioperative Medicine, The First Affiliated Hospital of Zhengzhou University, Zhengzhou, China

**Keywords:** maternal death, predictor, pregnancy, pulmonary hypertension, feature importance

## Abstract

**Background:**

Previous studies have suggested that pregnant women with pulmonary hypertension (PH) have high maternal mortality. However, indexes or factors that can predict maternal death are lacking.

**Methods:**

We retrospectively reviewed pregnant women with PH admitted for delivery from 2012 to 2020 and followed them for over 6 months. The patients were divided into two groups according to 10-day survival status after delivery. Predictive models and predictors for maternal death were identified using four machine learning algorithms: naïve Bayes, random forest, gradient boosting decision tree (GBDT), and support vector machine.

**Results:**

A total of 299 patients were included. The most frequent PH classifications were Group 1 PH (73.9%) and Group 2 PH (23.7%). The mortality within 10 days after delivery was 9.4% and higher in Group 1 PH than in the other PH groups (11.7 vs. 2.6%, *P* = 0.016). We identified 17 predictors, each with a *P*-value < 0.05 by univariable analysis, that were associated with an increased risk of death, and the most notable were pulmonary artery systolic pressure (PASP), platelet count, red cell distribution width, N-terminal brain natriuretic peptide (NT-proBNP), and albumin (all *P* < 0.01). Four prediction models were established using the candidate variables, and the GBDT model showed the best performance (F1-score = 66.7%, area under the curve = 0.93). Feature importance showed that the three most important predictors were NT-proBNP, PASP, and albumin.

**Conclusion:**

Mortality remained high, particularly in Group 1 PH. Our study shows that NT-proBNP, PASP, and albumin are the most important predictors of maternal death in the GBDT model. These findings may help clinicians provide better advice regarding fertility for women with PH.

## Introduction

Pulmonary hypertension (PH) is a pathophysiological disorder characterized by proliferation, narrowing, and remolding of the pulmonary vasculature and can complicate respiratory and cardiovascular diseases, which lead to right heart failure and premature death (1). The estimated 5-year survival rate is 72% in highly functioning patients and as low as 28% for those presenting with advanced symptoms (2).

Compared to men, women are two to four times more common to develop PH (3, 4). Moreover, women affected by PH are often young and in their childbearing age (4, 5). The maternal mortality rate for PH in pregnancy is known to be high (16–30%) (6). That is largely because of extensive physiological changes during pregnancy, such as an increase in intravascular volume, red cell mass, coagulability, oxygen consumption, cardiac output and a decrease in systemic vascular resistance, which may contribute to right ventricular failure (7). Most deaths occur during delivery or within 10 days after delivery, mainly due to right heart failure and cardiovascular collapse (4, 8–10). Therefore, the current guidelines recommend that pregnancy should be avoided in women with PH, especially those with pulmonary arterial hypertension (PAH) (1, 4, 6). However, some women develop this condition during pregnancy. In addition, socioeconomic, religious, or cultural factors drive some women with PH to desire to have a child. For these reasons, they decide to become pregnant or continue with an unplanned pregnancy (4, 8).

To date, due to limitations involving small sample sizes for statistical comparisons in previous studies, the risk factors for maternal death in pregnancy with PH remain unclear (8, 9, 11, 12).

Therefore, an in-depth understanding of the risk factors may help to identify high-risk pregnancy and provide appropriate medical advice, including pre-pregnancy counseling, and optimal management during pregnancy, which would be particular useful for women with mild PH. Hence, in the present study, we aimed to analyze pregnancy outcomes, establish death-related predictive models, and screen death-related predictors among pregnant women with PH.

## Materials and Methods

### Study Patients

**FIGURE 1:**
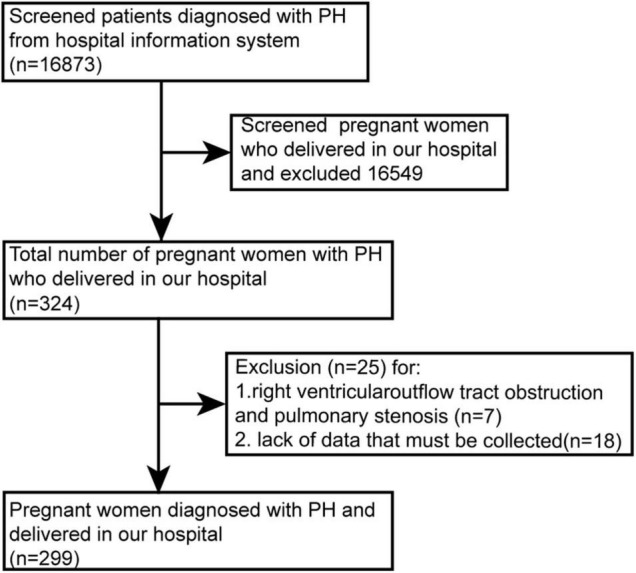
Patient flow chart. PH, pulmonary hypertension.

From June 2012 to December 2020, we retrospectively reviewed women with PH admitted for delivery in our hospital. Patients less than 18 years old, lacking necessary data (e.g., echocardiography, World Health Organization functional class), or with elevated right ventricular systolic pressure caused by outflow tract obstruction/pulmonary stenosis were excluded (Figure 1).

The diagnostic criterion for PH was a mean pulmonary arterial pressure ≥ 25 mmHg at rest measured by right heart catheterization (RHC) (1). In addition, if RHC was not available for the echocardiographic criteria for intermediate or high probability of PH, tricuspid regurgitation velocity > 2.8 m/s or tricuspid regurgitation velocity ≤ 2.8 m/s combined with at least two different categories of other echocardiographic signs (detail criteria see Supplementary Table 1) was also acceptable in the present study (1). A pregnancy loss occurring before 24 weeks of gestation was defined as abortion (13).

### Data Collection

We collected data from the Hospital Information System. Baseline data included demographic characteristics, diagnostic methods, diagnostic time, prior medical status, pregnancy history, gestational age, and PH etiology. Additionally, data on management and pregnancy outcomes, including delivery mode, anesthesia method, neonatal sex, birth weight, medication after delivery, and maternal or fetal vital status, were collected. Furthermore, the following risk factors and outcome predictors reported for PH and pregnancy in previous studies were collected, including the presence or absence of clinical signs of right heart failure, the progression of symptoms, syncope, World Health Organization (WHO) functional class, right atrium (RA) area, pericardial effusion, Eisenmenger syndrome, pulmonary artery systolic pressure (PASP), complications (e.g., peripartum cardiomyopathy, preeclampsia, diabetes mellitus), and laboratory parameters such as platelet count, red cell distribution width (RDW), lymphocyte count, neutrophil-to-leukocyte ratio, N-terminal brain natriuretic peptide (NT-proBNP), prothrombin time (PT), activated partial thromboplastin time (APTT), thrombin time, plasma fibrinogen, creatinine, blood urea nitrogen (BUN), uric acid, serum albumin, and globulin (1, 4, 5, 14–17). The clinical features of right heart failure, WHO functional class and progression of symptoms were defined according to the guidelines (1). The RA area, pericardial effusion, presence of Eisenmenger syndrome, and PASP were measured by echocardiography. All collected data from laboratory measurements and echocardiography were evaluated within 1 week before delivery in our center. Survival status was obtained by hospital records or telephone interviews. Based on the survival status within 10 days after delivery, patients were divided into two groups (survivors and non-survivors).

### Statistical Analysis

Univariable analysis: (a) Normally distributed continuous variables are presented as the mean ± *SD* (standard deviation) and were analyzed using Student’s *t*-test. (b) Non-normally distributed variables are presented as the median with the first and third quartiles: M (Q1, Q3) and were analyzed by Mann-Whitney *U*-tests. (c) Categorical variables are presented as numbers and percentages, and comparisons of groups were performed using Pearson’s chi-square tests with a theoretical frequency ≥ 5. (d) Continuity adjusted chi-square tests were used for theoretical frequency ≥ 1 but < 5. (e) Fisher’s exact tests were used for theoretical frequency < 1. The above statistical analyses were performed using IBM SPSS version 25.

Four machine learning (ML) algorithms—random forest (RF), naïve Bayes (NB), gradient boosting decision tree (GBDT), and support vector machine (SVM)—were employed to establish death-related prediction models and detect critical predictors in the present study. In some real-world studies, the classified data might have a bias because of class imbalance. Some outcomes as minority classes for binary classification were rare events, while other outcomes referred to as majority classes were commonly occurring events (18). The imbalance ratio (the ratio of the minority class size to the majority class size) of the datasets was above10:1, which could be regarded as a highly imbalanced dataset (18). This issue would decrease the predictive performance of the classifiers in machine learning (18). The proportions of survivors (90.6%) and non-survivors (9.4%) were also imbalanced in our study. The synthetic minority oversampling technique (SMOTE) algorithm employed in our study, which generates minority samples in the training dataset before training, is one of the best-known oversampling techniques, is most commonly applied to handle class imbalance problems and improve the predictive performance of models (18). Variables with *P* < 0.05 in the univariable analyses were included in the models. With the random state, the original dataset was randomly divided into a training dataset and a test dataset at a ratio of 8:2.

Evaluation of the model’s stability and parameter tuning were performed *via* an internal K-fold cross-validation (*k* = 5) in the training set after SMOTE. We adopted the accuracy, recall, precision, fuzzy measure (F1-score), and area under the curve (AUC) and mainly used the AUC and F1-score of the independent tested dataset to evaluate the performance of the models (19). Precision = $\frac{\text{TP}}{\text{TP}+\text{FP}}$, Recall = Sensitivity = $\frac{\text{TP}}{\text{TP}+\text{FN}}$, Accuracy = $\frac{\text{TP}+\text{TN}}{\text{P}+\text{N}}$ (TP: true positives, FP: false positives, FN: false negatives, P: positives, N: negatives). The F1-score is a weighted harmonic means of precision and recall. Feature importance for the best-performing model was reported to screen key predictors (20). The ranking of feature importance is based on information gain, which is employed to evaluate the additional information provided by each feature to the classifiers. Building and assessment of the prediction model, SMOTE, of the training dataset was performed with Python 3.6 (Python Software Foundation) using the scikit-learn library. Furthermore, the cut-off levels of significant predictors to predict death among pregnant women with PH could be found based on the receiver operating characteristic (ROC) curve using IBM SPSS version 25.

## Results

### General Demographic and Clinical Characteristics

This study included 299 patients (Figure 1). PH was diagnosed in 27 (9.0%) patients by RHC. The other 272 (81.0%) cases were diagnosed by echocardiography, of which 41 (13.7%) cases had RHC for reconfirmation after delivery, mostly during congenital heart disease surgery. Sixty-six patients (22.1%) were diagnosed before pregnancy, while 233 patients (77.9%) were newly diagnosed during pregnancy. Among the 233 cases, some developed PH only during pregnancy because the pre-pregnancy check-ups did not identify PH, the other did not know that PH was present before pregnancy.

The median age of the 299 patients was 28 years (Q1–Q3 = 25.0–31.0). The median gestational age was 35.6 weeks (Q1–Q3 = 29.7–38.1). The median PASP was 57.0 mmHg (Q1–Q3 = 42.0–87.0). One hundred fifty-one (51.8%) of them were nulliparous. Further classification of the 299 cases showed that 221 cases (73.9%) displayed Group 1 PH (PAH). Of these women, 24 (10.9%) had idiopathic PAH. Nineteen women (8.6%) had connective tissue disease-associated pulmonary arterial hypertension. One hundred seventy-eight patients (80.5%) had congenital heart disease associated with pulmonary arterial hypertension (atrial septal defect, *n* = 81; ventricle septal defect, n = 79; patent ductus arteriosus, n = 15; tetralogy of Fallot, *n* = 3). Seventy-one (23.7%) patients had Group 2 PH (due to left heart disease), with rheumatic heart disease in 39 (13.0%) women and cardiopathy in 32 (10.7%) women. Three patients (1.0%) had Group 3 PH (due to lung disease and/or hypoxia), and four (1.3%) patients had Group 5 PH (with unclear and/or multifactorial mechanisms).

Among these cases, 17 (5.7%) patients had twin pregnancies, and 47 (15.7%) patients (less than 24 weeks of gestation) underwent assisted abortion, including therapeutic abortion, curettage and cesarean section. The patients gave birth to a total of 259 neonates, of which 82 (31.7%) had birth weights below 2,500 g, and 30 (11.6%) died during the neonatal period. Thirty (11.6%) fetal or neonatal deaths up to 10 days after delivery occurred in 26 (8.7%) pregnancies, including 4 (1.3%) twin pregnancies. Additional clinical characteristics are presented in Table 1.

**TABLE 1:** Comparison of demographic and clinical characteristics between the survivor and non-survivor groups.

	Total patient (*n* = 299)	Survivors (*n* = 271)	Non-survivors (*n* = 28)	t/z/χ^2^	*P* *
**Characteristics before delivery**					
Age (years)	28.0 (25.0, 31.0)	28.0 (25.0, 31.0)	28.0 (24.0, 31.0)	-0.642	0.521
Gestational age (weeks)	35.6 (29.7, 38.1)	35.9 (29.7, 38.1)	31.9 (28.7, 36.7)	-2.025	0.043
PH diagnosis before pregnancy, *n* (%)	66 (22.1)	57 (21.0)	9 (32.1%)	1.821	0.177
Nulliparous, *n* (%)	155 (51.8)	140 (51.7)	15 (53.6)	0.037	0.847
Clinical classification of PH, *n* (%)				5.750	0.016^†^
Group 1	221 (73.9)	195 (72.0)	26 (92.9)		
IPAH	24 (8.0)	20 (7.4)	4 (14.3)		
CTD-PAH	19 (6.4)	17 (6.3)	2 (7.1)		
CHD-PAH	178 (59.5)	158 (58.3)	20 (71.4)		
Other groups of PH	78 (26.1)	76 (28.0)	2 (7.1)		
Group 2	71 (23.7)	69 (25.5)	2 (7.1)		
Group 3	3 (1.0)	3 (1.1)	0 (0.0)		
Group 5	4 (1.3)	4 (1.5)	0 (0.0)		
Right heart failure, *n* (%)	70 (23.4)	55 (20.3)	15 (53.6)	15.672	0.001
Progression of symptoms, *n* (%)	156 (52.2)	134 (49.4)	22 (78.6)	8.627	0.003
WHO functional class, *n* (%)				24.582	0.001
I, II	150 (50.2)	145 (53.5)	5 (17.9)		
III	96 (32.1)	87 (32.1)	9 (32.1)		
IV	53 (17.7)	39 (14.4)	14 (50.0)		
Pericardial effusion, *n* (%)	48 (16.1)	43 (15.9)	5 (17.9)	0.075	0.785
RA area (cm^2^)	19.5 (14.7, 25.0)	19.4 (14.5, 24.6)	22.5 (18.9, 28.2)	-2.777	0.005
PASP (mm Hg)	57.0 (42.0, 87.0)	55.0 (41.0, 80.0)	95.5 (76.0, 111.5)	-5.005	0.001
Disease-targeted therapies during pregnancy^‡^, *n* (%)	20 (9.0)	17 (8.7)	3 (11.5)	0.014	0.905
Comorbidity, *n* (%)	68 (22.7)	65 (24.0)	3 (10.7)	2.544	0.111
Eisenmenger syndrome, *n* (%)	47 (15.7)	38 (14.0)	9 (32.1)	4.997	0.025
Platelet count (10^3^/μL)	184.8 ± 75.1	191.2 ± 71.9	120.4 ± 76.0	-4.957	0.001
Lymphocyte count (10^3^/μL)	1.5 (1.2, 1.9)	1.5 (1.2, 1.9)	1.5 (1.2, 1.9)	0.169	0.866
Neutrophil-to-leukocyte ratio	4.2 (3.2, 6.0)	4.1 (3.1, 5.8)	4.9 (3.3, 7.8)	-1.743	0.081
RDW (%)	14.5 (13.7, 16.6)	14.4 (13.6, 16.5)	15.5 (14.5, 17.0)	-2.595	0.009
NT-proBNP (pg/mL)	366.2 (122.9, 1165.8)	314.1 (103.3, 1165.8)	2255.0 (786.2, 3619.0)	-5.105	0.001
Thrombin time (s)	13.9 (12.9, 15.2)	13.9 (12.9, 15.3)	13.9 (13.0, 15.2)	-0.242	0.809
PT (s)	9.8 (9.2, 10.5)	9.7 (9.2, 10.4)	10.3 (9.8, 11.5)	-2.943	0.003
APTT (s)	29.3 (27.2, 32.4)	29.3 (27.1, 32.2)	31.2 (29.3, 34.6)	-2.472	0.013
Fibrinogen (g/L)	3.5 ± 0.8	3.5 ± 0.8	3.3 ± 1.0	-1.357	0.176
Creatinine (μmol/L)	49.0 (42.3, 61.0)	49.0 (42.0, 60.0)	53.5 (46.3, 71.8)	-1.867	0.062
BUN (mmol/L)	3.8 (3.1, 4.9)	3.8 (3.0, 4.8)	5.0 (3.5, 7.4)	-2.998	0.003
Uric acid (μmol/L)	328.0 (255.0, 423.0)	323.0 (253.0, 414.0)	426.0 (330.0, 576.5)	-3.435	0.001
Albumin (g/L)	32.6 ± 4.9	32.9 ± 4.8	30.3 ± 5.2	-2.681	0.007
Globulin (g/L)	29.5 (26.7, 32.4)	29.5 (26.6, 32.3)	29.6 (27.1, 33.0)	-0.497	0.619
**Management**					
Abortion, *n* (%)	47 (15.7)	42 (15.5)	5 (17.9)	0.003	0.957
Anesthesia methods				9.922	0.007
General anesthesia, *n* (%)	115 (38.5)	97 (35.8)	18 (64.3)		
Neuroaxonal anesthesia, *n* (%)	156 (52.2)	149 (49.8)	7 (25.0)		
No anesthesia	28 (9.4)	25 (9.2)	3 (10.7)		
Vaginal delivery, *n* (%)	53 (17.7)	47 (17.3)	6 (21.4)	0.078	0.780
Disease-targeted therapies after delivery^‡^, *n* (%)	65 (29.4.)	55 (20.3)	10 (35.7)	1.162	0.281

*P-value of the difference between the survivor group and the non-survivor group, ^†^P-value for Group 1 and other groups, ^‡^not applicable in 78 cases of other group PH. PH, pulmonary hypertension; IPAH, idiopathic pulmonary arterial hypertension; CTD-PAH, connective tissue disease-associated pulmonary arterial hypertension; WHO, World Health Organization; RA, right atrium; PASP, pulmonary artery systolic pressure; RDW, red cell distribution width; NT-proBNP, N-terminal brain natriuretic peptide; PT, prothrombin time; APTT, activated partial thromboplastin time; BUN, blood urea nitrogen.

### Maternal Mortality and Characteristics of the Survivors and Non-survivors

Twenty-eight (9.4%) women died within 10 days after delivery. The median time of death was 1 day after delivery (ranging from during delivery to 8 days). Of the patients who died, 27 died of heart failure, and one died of postpartum hemorrhage. Sixteen neonates with a gestational age over 24 weeks survived among 23 cases with maternal deaths. Three (1.3%) of the 233 patients died within 6 months after delivery (one at 1 month, one at 3 months, and another at 5 months after delivery). Two (0.7%) patients had syncope, and 20 (6.7%) patients took anticoagulants before delivery. Information on the management of the PH patients is presented in Table 1.

The median gestational age of the survivor group was 35.9 weeks (Q1–Q3 = 29.7–38.1), and the mean gestational age of the non-survivor group was 31.9 weeks (Q1–Q3 = 28.7–36.7). We found that the mortality was higher for the women with Group 1 PH (26 of 221 cases, 11.7%) than for those with other types of PH (2 of 78 cases, 2.6%) (*P* = 0.016). The mortality rate differed significantly between women with and without Eisenmenger syndrome [19.1% (9 of 47 cases) vs. 7.5% (19 of 252 cases), *P* = 0.025]. Compared to the survivor group, the non-survivor group had a worse WHO functional class (I, II class: 17.9% vs. 53.5% and IV class: 50.0% vs. 14.4%, *P* = 0.001). The incidences of right heart failure (53.6% vs. 20.3%, *P* = 0.001) and progression of symptoms (78.6% vs. 49.4%, *P* = 0.003) were higher in the non-survivor group than in the survivor group. Patients in the non-survivor group had a larger RA area [23.9 ± 6.9 cm^2^ vs. 19.4 (14.5, 24.6) cm^2^, *P* = 0.005], higher PASP [92.7 ± 8.0 mmHg vs. 55.0 (41.0, 80.0) mmHg, *P* = 0.001], lower platelet count [(120.4 ± 76.0) × 10^3^/μL vs. (191.2 ± 71.9) × 10^3^/μL, *P* = 0.001], higher RDW [15.5% (14.5, 17.0) vs. 14.4% (13.6, 16.5), *P* = 0.009], higher NT-proBNP [2255.0 (786.2, 3619.0) pg/ml vs. 314.1 (103.3, 1165.8) pg/mL, *P* = 0.001], longer PT [10.3 (9.8, 11.5) s vs. 9.7 (9.2, 10.4) s, *P* = 0.003], longer APTT [31.2 (29.3, 34.6) s vs. 29.3 (27.1, 32.2) s, *P* = 0.013], higher BUN [5.5 ± 2.4 mmol/L vs. 3.8 (3.0, 4.9) mmol/L, *P* = 0.029] and higher uric acid [439.7 ± 142.5μmol/L vs. 323.0 μmol/L (253.0, 414.0), *P* = 0.001], and lower ALB (30.3 ± 4.3 g/L vs. 33.1 ± 5.0 g/L, *P* = 0.039) than the patients in the survivor group. In a comparison of neuroaxonal anesthesia and no anesthesia, women under general anesthesia had the highest mortality (18/115, 15.7%), (*P* = 0.007) (Table 1).

### Performance and Feature Importance of Prediction Models

With the random state, the original dataset was randomly divided into a training dataset and a test dataset at a ratio of 8:2. (*n* = 239 in the training dataset, *n* = 60 cases in the test dataset). With the SMOTE, the training dataset samples increased to 434 (217 cases in the survivor group and 217 cases in the non-survivor group). In the test dataset, 54 cases were in the survivor group, and 6 cases were in the non-survivor group.

Feature selection was based on a *P*-value < 0.05 in univariable analyses. Fivefold cross-validation was performed on the training dataset after SMOTE. After parameter tuning in fivefold cross-validation, we built four prediction models based on the total training dataset by machine learning algorithms. Table 2 shows the comparisons of different algorithms for the cross-validation and the training group.

**TABLE 2:** Model performance of the training and test datasets.

	Precision (%)	Recall (%)	Accuracy (%)	F1-score (%)	AUC
**Cross-validation set**					
NB	85.2	62.3	75.1	71.2	0.77
GBDT	95.2	100.0	97.5	97.5	1.00
SVM	81.8	70.7	77.7	75.7	0.81
RF	97.1	100.0	98.40	98.5	1.00
**Training set**					
NB	84.6	60.8	74.9	70.8	0.76
GBDT	100.0	100.0	100.0	100.0	1.00
SVM	83.8	73.7	79.7	78.4	0.83
RF	100.0	100.0	100.0	100.0	1.00
**Test set**					
NB	33.3	33.3	86.7	33.3	0.83
GBDT	100.0	50.0	95.0	66.7	0.93
SVM	57.1	66.7	91.7	61.5	0.90
RF	75.0	50.0	93.3	60.0	0.94

*AUC, area under the curve; NB, naïve Bayes; GBDT, gradient boosting decision tree; SVM, support vector machine; RF, random forest.*

For the test dataset, the F1-score, precision, accuracy, recall, and AUC values of the models are outlined in Table 2. The GBDT model obtained the highest F1-score (66.7%), precision (100.0%), and accuracy (95.0%), with the second-highest recall (50.0%) and AUC (0.93). Therefore, the GBDT model performed best in this study.

**FIGURE 2:**
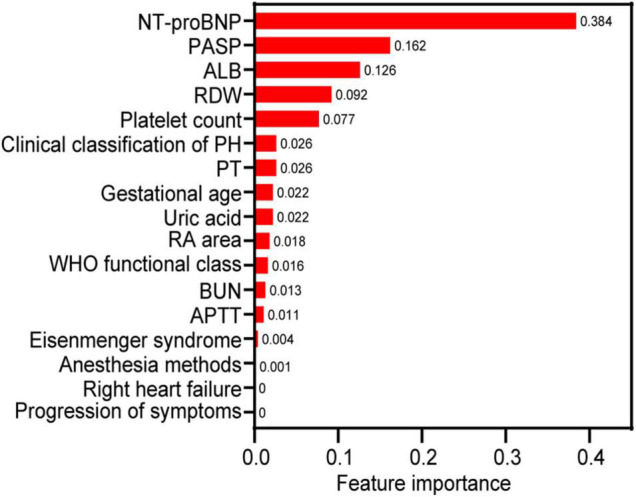
The relative feature importance of predictor variables included in the gradient boosting decision tree for predicting the death of pregnant women with pulmonary hypertension. NT-proBNP, N-terminal brain natriuretic peptide; PASP, pulmonary artery systolic pressure; ALB, albumin; RA, right atrium; APTT, activated partial thromboplastin time; RDW, red cell distribution width; PT, prothrombin time; WHO, World Health Organization.

The correlations between the variables are shown in Supplementary Figure 1. The WHO functional class was moderately and positively correlated with right heart failure and progression of symptoms, and BUN was moderately and positively correlated with uric acid (all r > 0.5, *P* < 0.05). Each variable’s feature importance from the GBDT model showed that the top three most critical predictive variables were NT-proBNP, PASP, and ALB, and is shown in Figure 2 after normalization processing (score for each feature importance = $\frac{\mathrm{the}\,\mathrm{raw}\,\mathrm{score}\,\mathrm{for}\,\mathrm{each}\,\mathrm{feature}\,\mathrm{importance}}{\mathrm{the}\,\mathrm{sum}\,\mathrm{of}\,\mathrm{all}\,\mathrm{raw}\,\mathrm{scores}\,\mathrm{for}\,\mathrm{features}\,\mathrm{importance}}$). The order of feature importance across fivefold cross-validation runs was also analyzed, and this was stable and similar (Supplementary Table 2). The ranking of feature importance is based on information gain, which is employed to evaluate the additional information provided by each feature to the classifiers.

**FIGURE 3:**
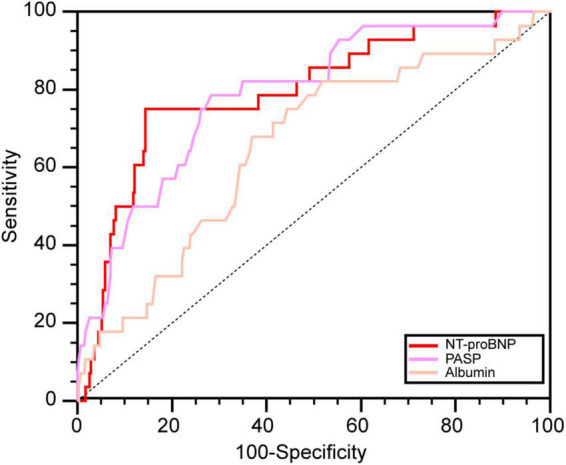
The ROC curves of significant predictors. NT-proBNP, N-terminal brain natriuretic peptide; PASP, pulmonary artery systolic pressure; ALB, albumin.

The areas under the ROC curves for NT-proBNP, PASP, and ALB to predict death in pregnant women with PH were 0.793, 0.787, and 0.654, respectively (all *P* < 0.05) (Figure 3). Based on the Youden index, the optimal cut-off values of NT-proBNP, PASP, and ALB were 1519.3 pg/mL, 74.0 mmHg, and 31.7 g/L, with sensitivity and specificity values of 75.0 and 85.6%, 78.6 and 71.6%, and 67.9 and 63.1%, respectively.

## Discussion

Our study has a unique large sample, focusing on pregnancy outcomes and including 299 pregnant women with PH, compared to other studies (5, 8, 10, 11). This is a scarce original study that sought to identify predictors for maternal death in pregnancies with PH by examining demographic and clinical characteristics, and the predictors mainly included NT-proBNP, PASP, ALB, RDW, and platelet count (21). Based on these variables, which were screened as predictors by univariable analysis, we first established a well-performing prediction model (the GBDT model) for maternal death. According to the feature importance in the GBDT model, we found that the three most important covariates were NT-proBNP, PASP, and ALB.

In our study, the maternal mortality within 10 days after delivery was 9.4% for all PH patients and 11.7% for PAH patients. The overall outcome was better than that reported 10 years ago (mortality 30–56%) (21). Recent studies by Karen and Marie-Louise reported 10-day mortality rates for PH (3.3–12.2%) and PAH (5.1–16.7%) that were similar to those in our study (8, 9). The reduction in maternal mortality in recent reports reflected advanced medical monitoring during the perinatal period. Consistent with other studies, the most common causes of death were right ventricular failure and shock, occurring at the early stage after delivery (8, 9, 11).

Machine learning in medicine has become a hot topic and is widely used to estimate risk, determine predictors and develop prediction models for diagnosis and prognosis with superior predictive ability (22). Machine learning has been used in the analysis of big data, but the four algorithms employed in this present study, namely, RF, NB, GBDT, and SVM, can also perform well with small sample sizes (23–25). For example, Galatzer-Levy et al. developed post-traumatic stress disorder predictive models by SVM with 152 samples (26); Lee Jollans reported that RF had good performance with a sample size over 200 and could perform across all sample sizes (23). Compared to traditional biostatistics, Machine learning can build models using datasets with more features exceeding the sample size (23).

In our prediction model, NT-proBNP was the most important predictor for maternal death. NT-proBNP, a natriuretic peptide, is released from cardiomyocytes due to ventricular stretch, and an elevated level in PH predicts overload of pressure and heart failure (15). NT-proBNP is also a serum biomarker that has strong predictive value for mortality in adult congenital heart disease (27, 28) and is recommended for risk assessment in PH patients by international guidelines (1). Pregnancy is accompanied by complex hemodynamic changes, including an increase in intravascular volume and cardiac output and a decrease in systemic vascular resistance, affecting the level of natriuretic peptides (29). However, clinical data also revealed that measurement of NT-proBNP had clinical utility in the risk assessment for pregnant women with cardiovascular disease (29, 30). Our study also found that the levels of NT-proBNP had utility in risk assessment and could predict death with a cut-off value of 1519.3 pg/ml and also had a good relative sensitivity and specificity for predicting death during delivery and within 10 days after delivery.

According to the model’s feature importance, PASP is another important predictor for high-risk patients and had relatively high sensitivity and specificity in further ROC analysis. The mean PAP and PASP values are not recommended as predictors of risk assessment in the guidelines, mainly because they will decrease with the reduction in stroke volume in the disease’s final stage (31). However, in the early or middle phase of the disease, stroke volume shows very little change. Furthermore, pulmonary artery pressure is central to evaluating disease progression and right ventricular dysfunction. An elevated PASP can indicate a high risk for ventricular dysfunction and progression of the disease in the early and middle stages (31, 32). Therefore, there may be two reasons to explain why PASP is a predictor of pregnancy risk in women with PH. First, stroke volume increases during pregnancy, unlike the decline in the final stage of PH (29). Second, the large proportion of women in our study who were asymptomatic and newly diagnosed with PH with WHO functional class I or II were in the early stage of the disease. In one Indian study and another East China study, univariable analysis revealed that maternal mortality increased in women with PASP > 70 mmHg and PASP > 50 mmHg, consistent with our study (5, 33).

In our study, serum albumin was also an important predictor for maternal death, and non-survivors had lower serum albumin, which is related to albumin’s biological functions. Albumin plays a vital role in numerous physiological processes, including antithrombotic functions, vascular endothelial stabilization, antioxidants, colloid osmotic pressure maintenance, and microvascular integrity (34). These pathophysiologic processes are relevant for disease progression in patients with PH, and therefore, hypoalbuminemia may represent a non-specific risk marker of more advanced PH (16). Several studies have demonstrated that hypoalbuminemia is linked to reduced survival in the setting of PH or heart failure, which was consistent with our findings (16, 34).

Although the top three most critical features had no highly correlated features (all *r* < 0.5), there was a weak correlation between the Eisenmenger syndrome and PASP (*r* = 0.49). Women with Eisenmenger syndrome also had a higher maternal mortality rate in the present study. Therefore, we should also be aware that the low rank of Eisenmenger syndrome could be affected by the correlation. In addition, Eisenmenger syndrome, a binary variable, has the lower chance for high feature importance than continuous variables in the GBDT model. Therefore, we recommend that patients with Eisenmenger syndrome follow the current guidelines against pregnancy (35).

### Limitations

Our study has some limitations. According to the 2015 ESC/ERS guidelines, RHC is the gold standard for diagnosing PH, although Group 2 and 3 patients are not recommended to undergo RHC unless organ transplantation is considered (1). However, RHC is an invasive tool that was not recommended for routine monitoring during pregnancy by a previous study (8) or the Pulmonary Vascular Research Institute guidelines (4). Therefore, we employed both RHC and echocardiogram parameters as diagnostic criteria that were also employed in other studies (5, 8–10). Moreover, a meta-analysis revealed that echocardiography has high sensitivity and specificity (83 and 72%, respectively) for diagnosing PH (36). In addition, we excluded patients with right ventricular outflow tract obstruction/pulmonary stenosis to avoid the negative impact on the estimation of PASP and diagnosis of PH. Furthermore, it remains unclear whether the echocardiogram parameters for diagnosing PH will change with extensive physiological changing during pregnancy. Forty-one (13.7%) patients were diagnosed with PH by echocardiography before delivery; when RHC was performed after delivery, they were all reconfirmed as having PH in our study. The echocardiographic diagnosis is not the gold standard. Therefore, we consider echocardiography as a diagnostic method to be a limitation of our study.

## Conclusion

Maternal mortality remains high among women with PH, especially those with PAH. Our study demonstrates that NT-proBNP, PASP, and serum albumin levels are significant predictors of death among pregnant women with PH. These findings may help clinicians provide better advice on family planning for women of childbearing age with PH and provide timely and appropriate medical interventions.

## Data Availability Statement

The raw data supporting the conclusions of this article will be made available by the authors, without undue reservation.

## Ethics Statement

The studies involving human participants were reviewed and approved by the Institutional Review Board of The First Affiliated Hospital of Zhengzhou University. Written informed consent for participation was not required for this study in accordance with the national legislation and the institutional requirements.

## Author Contributions

ZC was the guarantor of submission and participated in the literature search and study design. L-LD, T-CJ, and P-FL participated in the study design, data collection, data analysis, and writing. XW, HS, and YW participated in the study design and figures. L-QJ, ML, and LA participated in data collection and data verification. X-GJ participated in data collection and data analysis. All authors contributed to the article and approved the submitted version.

## Conflict of Interest

The authors declare that the research was conducted in the absence of any commercial or financial relationships that could be construed as a potential conflict of interest.

## Publisher’s Note

All claims expressed in this article are solely those of the authors and do not necessarily represent those of their affiliated organizations, or those of the publisher, the editors and the reviewers. Any product that may be evaluated in this article, or claim that may be made by its manufacturer, is not guaranteed or endorsed by the publisher.

## Supplementary Material

Supplementary Figure 1 Correlation between variables. BUN, blood urea nitrogen; WHO; APTT, activated partial thromboplastin time; PT, prothrombin time; NT-proBNP, N-terminal brain natriuretic peptide; RDW, Red cell distribution width; PASP, pulmonary artery systolic pressure; RA, right atrium; World Health Organization; PH, pulmonary hypertension.

Supplementary Table 1 Echocardiographic signs suggesting pulmonary hypertension used to assess the probability of pulmonary hypertension in addition to tricuspid regurgitation velocity measurement.

Supplementary Table 2 Feature importance across 5-fold cross-validation.
