# Supplementary figures and images for: Predictors of Maternal Death Among Women With Pulmonary Hypertension in China From 2012 to 2020: A Retrospective Single-Center Study
Source: Front Cardiovasc Med. 2022 Apr 18;9:814557. doi: 10.3389/fcvm.2022.814557 (PMC9058072; doi:10.3389/fcvm.2022.814557)

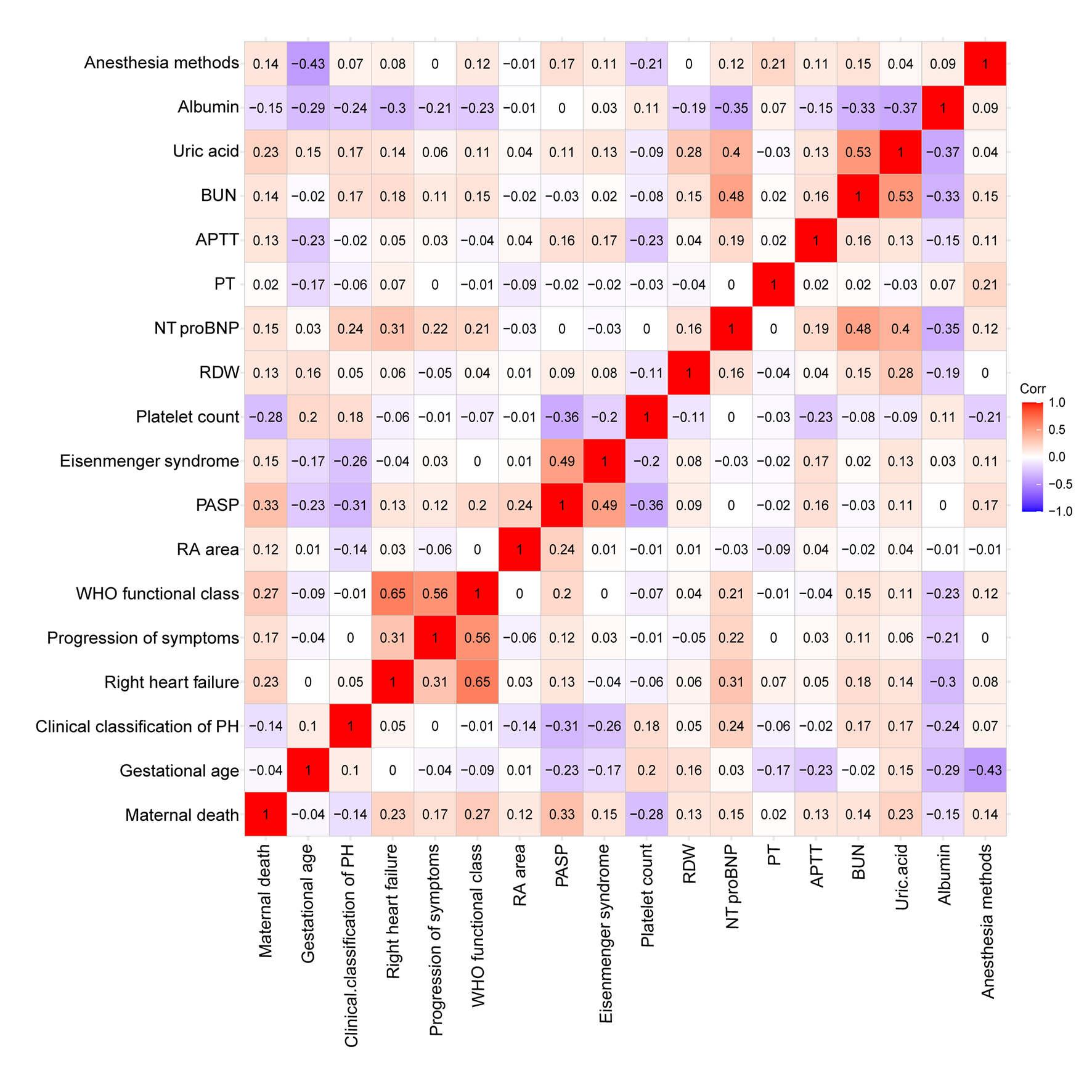

Supplement: Supplementary Figure 1 — Correlation between variables. BUN, blood urea nitrogen; WHO; APTT, activated partial thromboplastin time; PT, prothrombin time; NT-proBNP, N-terminal brain natriuretic peptide; RDW, Red cell distribution width; PASP, pulmonary artery systolic pressure; RA, right atrium; World Health Organization; PH, pulmonary hypertension. [file Image_1.TIF]
