# Supplementary material for: Predictors of Maternal Death Among Women With Pulmonary Hypertension in China From 2012 to 2020: A Retrospective Single-Center Study
Source: Front Cardiovasc Med. 2022 Apr 18;9:814557. doi: 10.3389/fcvm.2022.814557 (PMC9058072; doi:10.3389/fcvm.2022.814557)
Supplement: Supplementary Table 1 — Echocardiographic signs suggesting pulmonary hypertension used to assess the probability of pulmonary hypertension in addition to tricuspid regurgitation velocity measurement. [file Table_1.DOCX]

Table S1^a^ Echocardiographic signs suggesting pulmonary hypertension used to assess the probability of pulmonary hypertension in addition to tricuspid regurgitation velocity measurement

| A: The ventricles ^b^ | B: Pulmonary artery ^b^ | C: Inferior vena cava and right atrium ^b^ |
| --- | --- | --- |
| Right ventricle/left ventricle basal diameter ratio >1.0 | Right ventricular outflow Doppler acceleration time <105 msec and/or midsystolic notching | Inferior cava diameter >21 mm with decreased inspiratory collapse (<50% with a sniff or <20% with quiet inspiration) |
| Flattening of the interventricular septum (left ventricular eccentricity index >1.1 in systole and/or diastole) | Early diastolic pulmonary regurgitation velocity >2.2 m/sec | Right atrial area (end-systole) >18 cm2 |
|  | Pulmonary artery diameter >25 mm. |  |

Note: ^a^ cited from the guidelines (Galie et al., 2015). ^b^ Echocardiographic signs from at least two different categories (A/B/C) from the list should be present to alter the level of echocardiographic probability of pulmonary hypertension

Table S2 Feature importance across 5-fold cross-validation

| variable | Model 1 | Model 2 | Model 3 | Model 4 | Model 5 | Average |
| --- | --- | --- | --- | --- | --- | --- |
| NT-proBNP | 0.333 | 0.334 | 0.455 | 0.480 | 0.346 | 0.390 |
| PASP | 0.167 | 0.199 | 0.123 | 0.176 | 0.145 | 0.162 |
| Albumin | 0.136 | 0.060 | 0.130 | 0.051 | 0.048 | 0.085 |
| RDW | 0.101 | 0.094 | 0.076 | 0.124 | 0.079 | 0.095 |
| Platelet count | 0.063 | 0.069 | 0.064 | 0.067 | 0.174 | 0.087 |
| Clinical classification of PH | 0.046 | 0.015 | 0.037 | 0.004 | 0.009 | 0.022 |
| PT | 0.040 | 0.062 | 0.020 | 0.015 | 0.028 | 0.033 |
| Gestational age | 0.035 | 0.026 | 0.018 | 0.014 | 0.029 | 0.024 |
| Uric acid | 0.024 | 0.028 | 0.023 | 0.014 | 0.035 | 0.025 |
| BUN | 0.020 | 0.019 | 0.005 | 0.011 | 0.020 | 0.015 |
| WHO functional class | 0.016 | 0.019 | 0.028 | 0.022 | 0.026 | 0.022 |
| RA area | 0.009 | 0.045 | 0.010 | 0.014 | 0.053 | 0.026 |
| APTT | 0.009 | 0.014 | 0.007 | 0.007 | 0.004 | 0.008 |
| Eisenmenger syndrome | 0.002 | 0.014 | 0.003 | 0.002 | 0.002 | 0.005 |
| Anesthesia methods | 0 | 0 | 0 | 0 | 0 | 0 |
| Progression of symptoms | 0 | 0.004 | 0 | 0 | 0.001 | 0.001 |
| Right heart failure | 0 | 0.001 | 0 |  | 0 | 0 |

Abbreviations: NT-proBNP, N-terminal brain natriuretic peptide; PASP, pulmonary artery systolic pressure; ALB, albumin; RA, right atrium; APTT, activated partial thromboplastin time; RDW, red cell distribution width; PT, prothrombin time; WHO, World Health Organization.
